# Supplementary material for: Changing Attitudes toward the COVID-19 Vaccine among North Carolina Participants in the COVID-19 Community Research Partnership
Source: Vaccines (Basel). 2021 Aug 17;9(8):916. doi: 10.3390/vaccines9080916 (PMC8402530; doi:10.3390/vaccines9080916)
Supplement: Supplementary file 1 [file vaccines-09-00916-s001.zip › vaccines-1240357-supplementary.pdf]

SUPPLEMENTAL INFORMATION

QUESTIONNAIRE

COVID-19 Vaccine Survey

Q1. Have you already been vaccinated against COVID-19, or do you intend to be vaccinated once you are eligible to receive it?

- o Yes
- o No
- o Unsure
- o Prefer not to answer.

Q2. If you responded "No" or "Unsure" please indicate why (select all that apply)

a. I already had COVID-19 and believe I am now protected.
b. I received the active vaccine in a COVID-19 vaccine trial.
c. I do not think COVID-19 is a significant problem.
d. I am not sure the vaccine works.
e. I am not sure the vaccine is safe.
f. I don't trust the healthcare system.
g. Prefer not to answer.

Questionnaire sent to participants of the survey to assess attitudes toward COVID-19 vaccination.

Questionnaire for follow-up survey to assess whether or not participants received the COVID-19 vaccine.

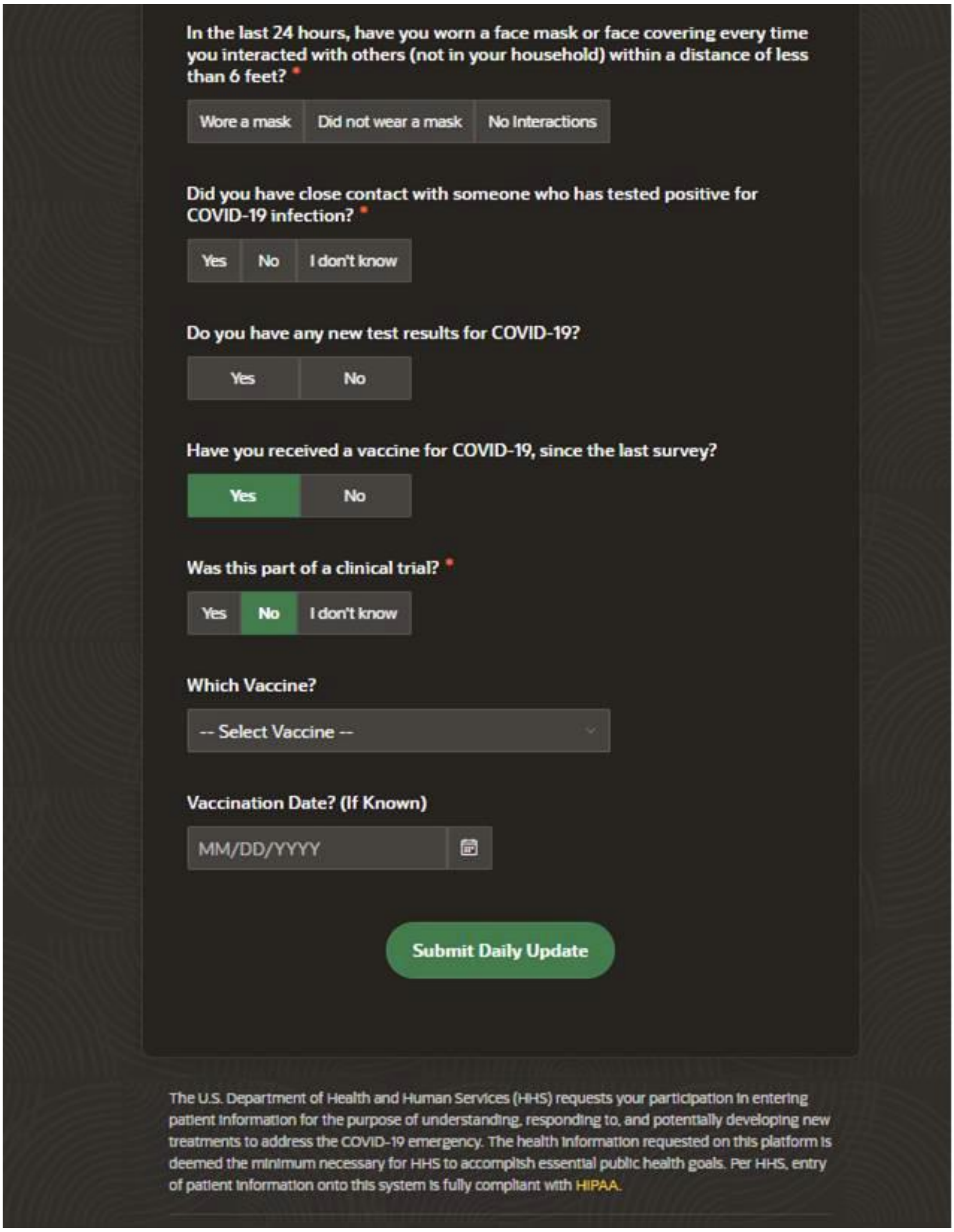

In the last 24 hours, have you worn a face mask or face covering every time you interacted with others (not in your household) within a distance of less than 6 feet? *

Wore a mask | Did not wear a mask | No Interactions

Did you have close contact with someone who has tested positive for COVID-19 infection? *

Yes | No | I don't know

Do you have any new test results for COVID-19?

Yes | No

Have you received a vaccine for COVID-19, since the last survey?

Yes | No

Was this part of a clinical trial? *

Yes | No | I don't know

Which Vaccine?

-- Select Vaccine --

Vaccination Date? (If Known)

MM/DD/YYYY

Submit Daily Update

The U.S. Department of Health and Human Services (HHS) requests your participation in entering patient information for the purpose of understanding, responding to, and potentially developing new treatments to address the COVID-19 emergency. The health information requested on this platform is deemed the minimum necessary for HHS to accomplish essential public health goals. Per HHS, entry of patient information onto this system is fully compliant with HIPAA.
